# Bacterial exo-polysaccharides in biofilms: role in antimicrobial resistance and treatments

**DOI:** 10.1186/s43141-021-00242-y

**Published:** 2021-09-23

**Authors:** Shivani Singh, Saptashwa Datta, Kannan Badri Narayanan, K. Narayanan Rajnish

**Affiliations:** 1 Department of Genetic Engineering, School of Bioengineering, SRM Institute of Science and Technology, Kattankulathur, Tamil Nadu India; 2 School of Chemical Engineering, Yeungnam University, 280, Daehak-Ro, Gyeongsan, Gyeongbuk 38541 Republic of Korea

**Keywords:** Biofilm, Exopolysaccharide, Drug resistance, Quorum sensing

## Abstract

**Background:**

Bacterial biofilms are aggregation or collection of different bacterial cells which are covered by self-produced extracellular matrix and are attached to a substratum. Generally, under stress or in unfavorable conditions, free planktonic bacteria transform themselves into bacterial biofilms and become sessile.

**Main body:**

Various mechanisms involving interaction between antimicrobial and biofilm matrix components, reduced growth rates, and genes conferring antibiotic resistance have been described to contribute to enhanced resistance. Quorum sensing and multi-drug resistance efflux pumps are known to regulate the internal environment within the biofilm as well as biofilm formation; they also protect cells from antibiotic attack or immune attacks. This review summarizes data supporting the importance of exopolysaccharides during biofilm formation and its role in antibiotic resistance.

**Conclusions:**

Involvement of quorum sensing and efflux pumps in antibiotic resistance in association with exopolysaccharides. Also, strategies to overcome or attack biofilms are provided.

## Background

Bacteria can produce a protective layer of a polymer during unfavorable conditions; it uses simple complex substrate to produce biopolymer of different characteristics. Microbial biofilm acts like a cooperative group made up of different microbial communities and shows structural as well as physiological complexity. Generally, the microbial biofilm develops on a substratum and is immersed in polymeric matrix that is secreted by the bacteria. Both auto and hetero-trophic microbes form stable biofilms for their survival in harsh conditions. They can develop in multiple environments such as sewage channels, bathrooms, laboratories, hospitals, biomedical equipment, hot springs, deep-sea vents, rivers, and rocks [1–3]. Biofilm-associated bacteria are implicated in broad range of infections such as respiratory tract infections in case of cystic fibrosis (CF), chronically infected wounds, and medical device related infections [4]. Biofilms was first described by Anton Von Leeuwenhoek during the seventeenth century as microbial aggregates on scrapings of plaque from his tooth. Bacterial exopolysaccharides (EPS) were initially discovered by Louis Pasteur in 1861, as a microbial product in wine and was named as a dextran. In 1878, Van Tieghem later discovered dextran-producing strain *Leuconostoc mesenteriodes.* In 1973, Characklis described these biofilms as persistent and resistant to disinfectant. The term “Biofilm” was coined by a Canadian microbiologist, Bill Costerton in 1978. He defined it as “a structured community of bacterial cells enclosed in a self-produced polymeric matrix and adherent to an inert or living surface” [2, 5, 6]. In 2002, Donlan and Costerton defined it as “a microbially derived sessile community characterized by cells that are irreversibly attached to a substratum or interface or each other, embedded in a matrix of extracellular polymeric substances that they have produced, and exhibit an altered phenotype concerning growth rate and gene transcription” [2, 5]. Biofilm of microbial colonies is encompassed by a bacterial extracellular matrix (ECM) that is composed of multiple types of exopolysaccharides, extracellular nucleic acids, and multiple types of proteins. These biofilms protect the bacteria from changes in pH, osmolarity, lack of nutrients, and various external mechanical forces. They also help in providing antibiotic resistance by blocking the access of antibiotics and immune cells of the host, and this property gives rise to drug resistance in bacteria. Biofilms are considered to be responsible for many of the chronic bacterial infections that take place. Most of the most commonly acquired bacterial nosocomial infections happen because various pathogenic bacteria from biofilms on catheters, hospital equipment, and surgical instruments [3, 7]. The construction of these biofilms is sometimes regulated by a certain mechanism known as quorum sensing. The bacteria communicate with other bacteria using signaling molecules called quorum sensing molecules. These molecules help regulate the expression of certain genes that help the bacterial community survive various conditions. In case of biofilms, it has been observed that these molecules help in the disassembly of communities in a biofilm [8–10]. Cellulose is an important polysaccharide that forms the backbone of biofilms of various bacteria like *Mycobacterium tuberculosis* [7]. Mostly microbial biofilms formed are elastic in nature due to the presence of a different flow of oxygen and nutrient within the biofilm. The structure and functions of a biofilm depend on the microbial colonies residing in it as well as the environmental conditions [2]. The basic functions of the biopolymers that are produced includes surface adherence, protection from protozoa and white blood cells (phagocytes) attack, and attack by antimicrobial agents. Biopolymers produced are differentiated based on chemical structure and composition, molecular weight, linkage bonds, and functions as well as the site at which they are produced. Depending on their site of origin, biopolymers are of two types: intracellular and extracellular [11]. There have been a multitude of mechanisms described in literature which are responsible for playing a major role in antibiotic resistance in bacterial infections such as limited diffusion of the antibiotics through the EPS matrix, phenotypic changes in response to antibiotic agents, and overexpression of efflux pumps. The entire repertoire of varying chemical molecules secreted by the bacteria and present on the cell surface is referred to as the matrixosome. The matrixosome is composed of secreted exopolysaccharides, proteins, a diverse group of proteins, and extracellular DNA and RNA. The complete matrixosome helps the bacteria to protect itself from drugs, stress, and helps it to improve its fitness, pathogenicity, and virulence [12]. There are multiple polysaccharides including cellulose, levans, alginates, pel, psl which perform architectural, protective, and aggregative functions in the biofilm [13]. The biofilm matrix of the pathogen *Pseudomonas aeuroginosa* is one of the most well studied. It can serve as a model for understanding biofilm formation in bacteria. The exopolysaccharides produced by these bacteria can help them in immune invasion, increased pathogenicity, and enhance their survival. In patients of cystic fibrosis, biofilm forming *Pseudomonas aeruginosa* isolates can cause life threatening infections [14]. Although there is a lot of literature explaining the composition of bacterial biofilms and its functions, there are limited manuscripts summarizing the role of these biofilms in antibiotic resistance and efficient treatments against these biofilms. In this review, we elucidate on the role of the EPS in providing resistance or tolerance to several antimicrobial agents and the role played by efflux pumps and QS in association with EPS [15] along with various strategies of treatment.

## Main text

**Fig. 1:**
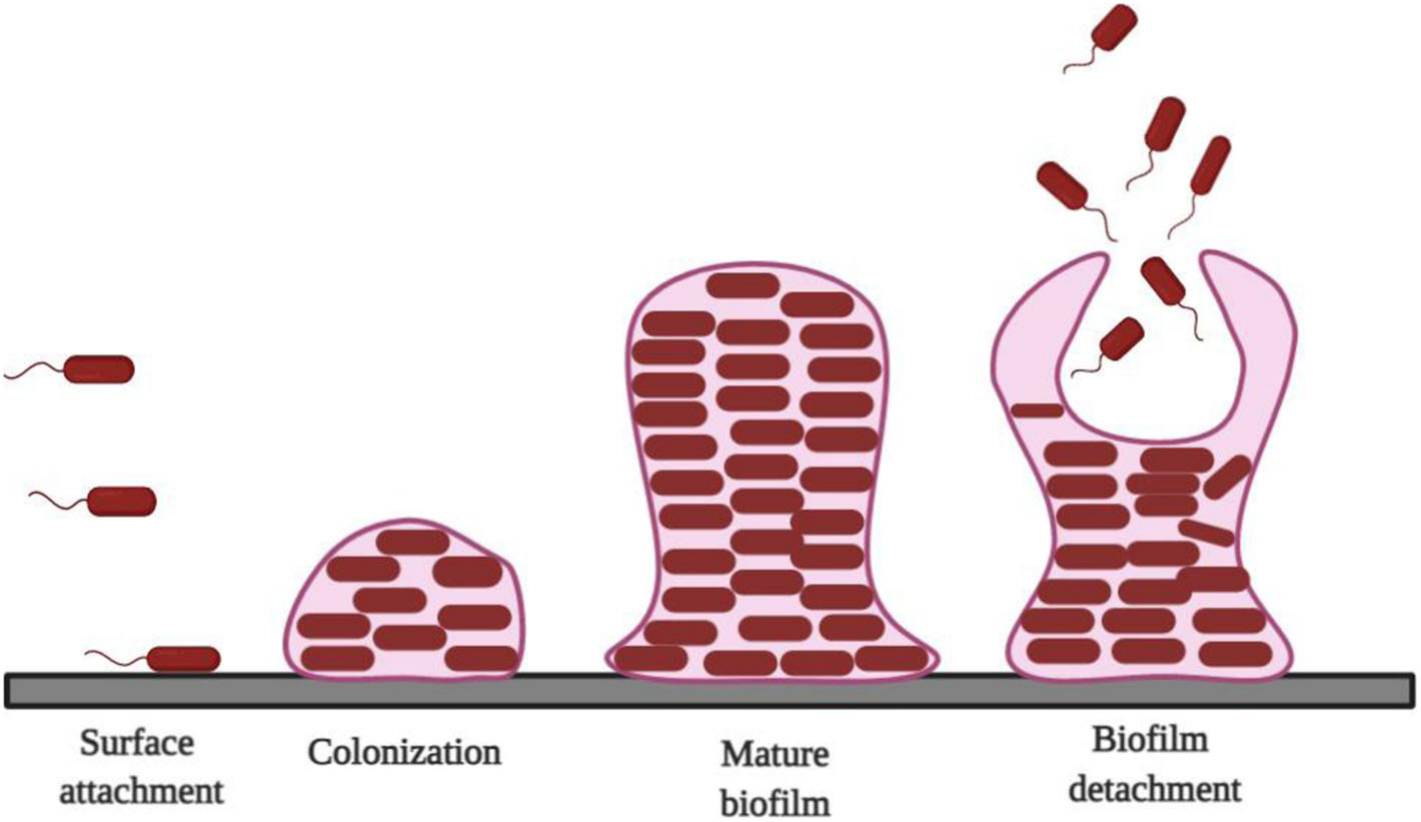
Diagrammatic representation of stages involved in biofilm formation. Stage 1: reversible attachment of bacterial cells to the surface; stage 2: production of the extracellular EPS matrix and early development of biofilm architecture; stage 3: maturation of biofilm architecture; stage 4: detachment bacterial biofilms.

Biofilm formation is a natural phenomenon, which occurs when the density of the population of bacterial cells reaches a certain threshold and so as a result leads to the formation of multiple micro-colonies. This process is further classified into four different phases, i.e., surface attachment, sessile growth phase which is controlled by intercellular interaction or colonization, biofilm maturation, and detachment [16–18] (Fig. 1).

### Biofilm attachment

This is the first and the important step that involves the affixing of the bacteria to a solid substratum, and is highly dynamic and reversible depending on the situation. Initially, bacteria are introduced to the surface where it is going to be attached. This process is majorly carried out by Brownian motion and gravitational forces and is also impacted by hydrodynamic forces in the vicinity of the bacteria. As it settles down on a substrate, microbe encounter attractive and repelling forces which may differ depending on nutrient levels, pH, ionic strength, temperature, and also the microbe cell-surface composition affects the biofilm attachment by regulating its velocity and direction. Motile bacteria have an advantage over non-motile bacteria since it can use flagella to bridle hydrodynamic forces and repulsive forces and that has been observed in *Pseudomonas aeruginosa*, *Vibrio cholerae*, *Listeria monocytogenes*, and *Escherichia coli*. Chemotaxis is also known to regulate biofilm attachment in some bacterial species. Adherence is further regulated by extracellular adhesive appendages and secreted adhesins. *P*. *aeruginosa* besides flagella uses type IV pili-mediated twitching motility to adhere to the substrate, and it also expresses chaperone usher pathway (CUP) fimbriae, among which CupA is involved in adherence and auto-aggregation. Similarly, *E*. *coli* relies on type I pili assembled by CUP for surface adherence in a niche-specific manner. In *E*. *coli* along with type I pili, curli fimbriae and Antigen 43 are known to regulate attachment and inter-bacterial interactions. In the case of non-motile bacteria such as *Enterococci*, adhesins such as SagA, Acm (*E*. *faecium*) and Ace (*E*. *faecalis*), and the surface protein Esp have been known to facilitate biofilm formation on abiotic surfaces [17].

### Growth phase

Once the bacteria get attached to the surface, the attachment induces the differential gene expression, upregulation of factors favoring attachment. Factors identified to support sessility are ECMs such as cellulose, polyglucosamine (PGA), and colanic acid which also contribute to the architecture. The composition of the extracellular matrix varies within bacterial species depending on the environmental conditions [17].

### Biofilm maturation

Maintaining biofilm attachment is important to regulate pathogenicity. CdrA is an adhesin secreted by *Pseudomonas species* in response to high levels of c-di-GMP signal, which further binds to polysaccharide synthesis locus (PSL) and helps to maintain the biofilm structures. Alginate, an EPS found in *P*. *aeruginosa*, its synthesis is regulated by a surface-bound diguanylate cyclase MucR through high local concentrations of c-di-GMP. Along with EPS, e-DNA has been observed to regulate cell to cell interactions and stabilizes *Pseudomonas* biofilm at initial stages*.* e-DNA is organized in a well-defined pattern and is located in the stalk part of the biofilms which were shaped like mushrooms [8].

### Biofilm detachment

Biofilm detachment can occur due to changes in the availability of nutrients, instability in concentrations of available oxygen levels, an elevation in levels of toxins in the vicinity, or due to conditions that induce stress in the bacteria [16]. Biofilm detachment mechanism is generally divided into two categories, i.e., active and passive. In active detachment, bacteria themselves initiate the process through enzymatic degradation, quorum sensing (QS), etc. Whereas passive detachment process involves external forces like fluid shear, abrasion, and human intervention [3] Biofilm detachment is an important area of medical research that deals with combating the [16] antibiotic susceptible-free dispersed bacteria and prevention of secondary biofilm formation. In the case of *P*. *aeruginosa* biofilms, detachment occurs due to increased carbon and nitrogen sources or due to action of the EPS degrading enzymes such as alginate lyase. Also, the void created within *P*. *aeruginosa* biofilms by cell death can facilitate biofilm detachment. In *E*. *coli*, the CsrA protein is known to inhibit PGA synthesis. The reduced c-di-GMP signal results in biofilm detachment in *P*. *aeruginosa* and *E*. *coli* [8]*.* Dispersing bacteria can form bacterial biofilm in suitable environmental conditions and thus result in chronic infections. So, this way it becomes difficult to treat biofilm-related infections.

## Quorum sensing

Quorum sensing (QS) is a cell-cell communication process that enables bacteria to modify their behavior in response to changes in the cell density, pH, signal flow rates, and surrounding microbial community [3, 19, 20]. It was discovered 25 years ago in two luminous marine bacterial species, *Vibrio fischeri* and *Vibrio harveyi*, and enzymes involved in QS were responsible for light production. The *lux* system of QS was first revealed in *V*. *fischeri*. Over 80% of bacterial species involved in biofilm formation exhibit this process. This process involves the production, release, and detection or recognition of extracellular signaling molecules called auto-inducers (AI) [18, 19, 21]. As the population cell density increases, AI accumulates in the extracellular environment and is detected by the bacterial cells present in the biofilms. This process has been described to control the secretion of virulence factors, the production of public goods, and the formation and maintenance of biofilms and also play role in bacterial interactions and the infection process [18, 20, 22]. Three types of QS mechanism have been described: (a) LuxI/LuxR-type QS in Gram-negative bacteria, (b) small peptides as signal molecules in Gram-positive bacteria, and (c) adopted by both Gram-negative and Gram-positive bacteria the LuxS-encoded auto-inducer-2 (AI-2). During the process, a signaling molecule is released and this secreted molecule binds to other recipient bacterial cells; this results in the activation of genes including genes responsible for the synthesis of these molecules [17].

The QS mechanism differs from Gram-negative bacteria to Gram-positive bacteria. The Gram-negative bacteria uses low-molecular-weight acyl derivatives of l-homoserine lactone (N-AHLs) as a chemical signal. Whereas in Gram-positive bacteria, chemical signals vary in nature which includes modified peptides, amino acids, and similar amino compounds bind to receptor histidine kinases [22]. Oligopeptides like thiolactone, furanone signals (LuxS system) in *V*. *harveyi* induced by AI-2, hydroxyl–palmitic acid in *Ralstonia solanacearum*, diketopiperazines in *P*. *aeruginosa*, methyl dodecanoic acid, and quinolone are examples of QS signaling molecules in Gram-negative bacteria. Gram-positive *Bacillus subtilis* and *Staphylococcus aureus* utilize eukaryotic-like Ser/Thr kinase-mediated regulation of biofilm formation [17].

### Gram-negative bacteria

In gram-negative bacteria, LuxI/LuxR-type QS mechanism has been described to induce virulence. During the process, AHLAI synthesized by LuxI homolog diffuses through the cell membrane, and at high concentration it binds and stabilizes the cognate LuxR receptor. This AHL-LuxR receptor protein complex results in an alteration in the expression of several genes while auto-inducing LuxI expression in feedforward reaction to produce more AHL AI to amplify the signal [10, 17]. QS information is generally integrated by small RNAs (sRNAs) that are known to control target gene expression and function in feedback loops. QS network architectures promote signaling fidelity, temporal control, and flexible input-output dynamics [18, 23].

### Gram-positive bacteria

In Gram-positive bacteria, there is a number of processes present that work in response to cell population density. In the absence of an outer membrane, the Gram-positive bacteria uses the cell wall as the scaffold for displaying a wide variety of surface molecules, which include teichoic acid, lipoteichoic acid, and several protein adhesins. Some of the surface proteins are bound to the cell wall non-covalently, and other proteins are anchored covalently to the peptidoglycan. Well-described QS system in Gram-positive bacteria includes competence for DNA uptake in *Bacillus subtilis* and *Streptococcus pneumoniae*, virulence in *S*. *aureus*, conjugation in *E*. *faecalis* and microcin production in *Lactobacillus sake* and *Carnobacterium piscicola*. Instead of LuxI/LuxR signaling circuit, Gram-positive bacteria consist of two-component signal transduction system (2CSTS); they secrete modified peptide signaling molecules that are usually dedicated by ABC (ATP-binding cassette) exporter protein. Secreted peptide signals are recognized by cognate two-component sensor kinase proteins that are known to interact with cytoplasmic response regulator proteins. Most of the sensor kinases found are membrane-bound proteins that auto-phosphorylate in the presence of ATP at a conserved histidine residue, from the phosphoryl are transferred to a conserved aspartate in the response regulator, thus this signal transduction mechanism is a phosphorelay cascade [10, 24]. Number of environmental factors such as pH, nutrition availability, and fluid flow and regulatory systems involving Sae, SarA, Rot are involved in the regulation of biofilm formation [25]. In the case of Gram-negative bacteria, we are going to discuss *S*. *aureus*, since it can cause a tremendous range of disease, from simple skin infections to life-threatening ailments, such as sepsis, endocarditis, and osteomyelitis.

Both flagella and twitching motility play an important role in the initiation of biofilm attachment. Motility is important for the cells to make initial contact with an abiotic surface. Type IV pili and type V pili mediate twitching mobility in *P*. *aeruginosa* biofilm development. Type IV pili plays a direct role in stabilizing interactions between the abiotic surfaces required to form a micro-colony and also helps the cell to migrate along the surface to form multi-cell aggregates. Strains defective in pili biogenesis expresses neither twitching motility nor micro-colony formation phenotypes. The *pilBCD* operon is thought to encode accessory factors required for type IV pili biogenesis [26].

## Role of efflux pumps in biofilm formation

Efflux pumps are membrane proteins responsible for the extrusion of substances from within the cell into the extracellular space. These pumps are found in both Gram-positive and Gram-negative bacteria as well as in eukaryotic organisms [18, 20, 27]. Efflux pumps is a key mechanism of cellular responses to various environmental factors and helps in regulating their internal environment by exporting toxic substances including antibiotic agents, metabolites, and QS signal molecules from within the cell [3, 15].

Bacterial efflux pumps are classified into five families depending on the number of components, number of transmembrane spanning regions, the energy source utilized for the export, and the types of molecules that are exported: MF (major facilitator) superfamily, MATE (multidrug and toxic efflux) superfamily, RND (resistance-nodulation-division) superfamily, SMR (small multidrug resistance) superfamily, PACE (proteobacterial antimicrobial compound efflux) superfamily, and ABC (ATP binding cassette) superfamily. The ABC family transporter makes use of ATP hydrolysis to stimulate the export of substances, whereas other family transporters make use of the proton motive force as an energy source. The expression of these pumps is tightly regulated [3, 18, 20, 27–31]. Efflux systems studied for antibiotic resistance in Gram-negative bacteria is RND superfamily and is composed of tripartite system consisting of cytoplasmic membrane pump, a periplasmic membrane fusion protein, and an outer membrane protein channel. This system allows direct removal of various antibiotics from cytoplasm or in some cases periplasmic space to the extracellular space [3, 4, 20, 28, 29]. In the case of Gram-positive bacteria, MFS and ABC superfamily efflux transporters are most commonly found [3, 18]. There are various efflux system that contribute to antibiotic resistance that have been discovered such as *Campylobacter jejuni* (CmeABC), *E*. *coli* (AcrAB-TolC, AcrEF-TolC, EmrB, EmrD), *P. aeruginosa* (MexAB-OprM, MexCD-OprJ, MexEF-OprN, and MexXY-OprM), *Streptococcus pneumoniae* (PmrA), *Salmonella typhimurium* (AcrB), and *S*. *aureus* (NorA) [27].

QS system are known to control biofilm formation by regulating expression of efflux genes. One such example is biofilm formation in *Burkholderia pseudomallei* dependent on BpeAB-OprB efflux pump [3] (Soto 2013). Also these efflux pumps export six different homoserine lactone signals which are involved in the QS response [31]. Overexpression of efflux system as consequences of mutations in the gene or in response to antibiotic agents provide resistance or tolerance to antibiotics [27]. Along with having an important role in biofilm development and EPS synthesis in different bacterial species, they also provide resistance to several antibiotics.

## Different mechanisms through which exopolysaccharide play a role in antibiotic resistance

The primary functions of EPS produced are to hold the bacterial community together, fix bacterial cells to solid surfaces, and to maintain proper hydration and nutrients availability. However, biofilms are considered one of the principal cause of chronic infections associated with indwelling medical devices such as catheters, surgical implants, also infections related to cystic fibrosis, osteomyelitis, rhino-sinusitis, and wound infections. EPS play a key role in the formation of biofilm and also in biofilm resistance to antimicrobial agents and host defense [32]. Biofilms protect bacteria from stresses like desiccation, oxidizing agents, and host immune responses [33]. Microbial biofilms use two principal ways to resist antibiotic treatment: genetically obtained antibiotic resistance and drug tolerance, and there is a difference between antibiotic resistance and drug tolerance. Antibiotic resistance can be acquired due to mutations in strains or mutations in resistance genetic determinants which increase the antimicrobial minimal inhibitory concentration (MIC) and as a result can resist the effect of antimicrobial agent. Genetic, environmental, and culture conditions influence the resistance gained by the bacterial species. Mechanisms such as increased oxidative stress, SOS responses to DNA damage, and RpoS-dependent responses result in mutation of genes in biofilm. Bacteria can also acquire resistance through horizontal gene transfer within the biofilm using e-DNA. The presence of multiple species of bacteria within the biofilm, proximity between the microbes, and highly hydrated matrix facilitates the horizontal gene transfer of genes encoding antibiotic resistance. Drug tolerance in another way is described as the ability of biofilm to resist antibiotic treatment without developing resistance mostly via persister cells and biofilm physiological states, and it can also be defined as phenotypic resistance [34–37].

Bacteria exhibit antibiotic resistance through various mechanisms, such as:

1. Decreased penetration of the antibiotics through polysaccharide matrix into the biofilm
2. Increased efflux of the antibiotics via efflux pumps or porins into the extracellular space
3. Phenotypic changes in the cells involved in biofilm formation
4. Presence of the persister cells deep in the biofilm
5. Regulation of internal environment by QS
6. Decreased growth rate in the core of the biofilm because of oxygen and nutrient gradient through the biofilm
7. Inactivation of antibiotic through modification such as phosphorylation by an enzyme
8. Alteration of the antibiotic target through post translational modification or any genetic mutation [3, 18]

There is not a common mechanism representing antibiotic resistance present, since the extracellular polymer matrix produced by the bacterial colonies differ as well as the environment surrounding them is different. These differences may result in adaptation of different mechanisms to survive the stressful conditions or antibiotic pressure.

### Reduced penetrance

One of the prominent ways by which bacterial biofilms resist antibiotics is through decreased penetration of antibiotics through the polysaccharide [6, 15, 38]. Formation of thick biofilm layer surrounding the bacterial species acts as both chemical and physical diffusion barrier to most of the antibiotics and thus retards the penetration of the antimicrobial or antibiotics into the biofilm. Penetration of antibiotics depend on thickness of the biofilms, diffusion efficacy of the agents, interaction of the agent with the biofilm, the sorption capacity of the biofilm for the agent, as well as the dose concentration and duration of the agent [39]. Binding of antibiotics to the biofilm component results in decreased antibacterial activity mostly by depletion of antibiotics. Sometimes, slow and limited penetration of antibiotics promote antibiotic tolerance by enabling antibiotic modifying enzymes such as β-lactamases to degrade aminoglycoside antibiotics [32, 33, 38]. Unlike planktonic *P*. *aeruginosa* cells, biofilm cells showed tolerance against hydrogen peroxide by restricting their penetrance in the biofilm and also result in destruction of the compound by the cells, thus providing resistance [38].

**Fig. 2:**
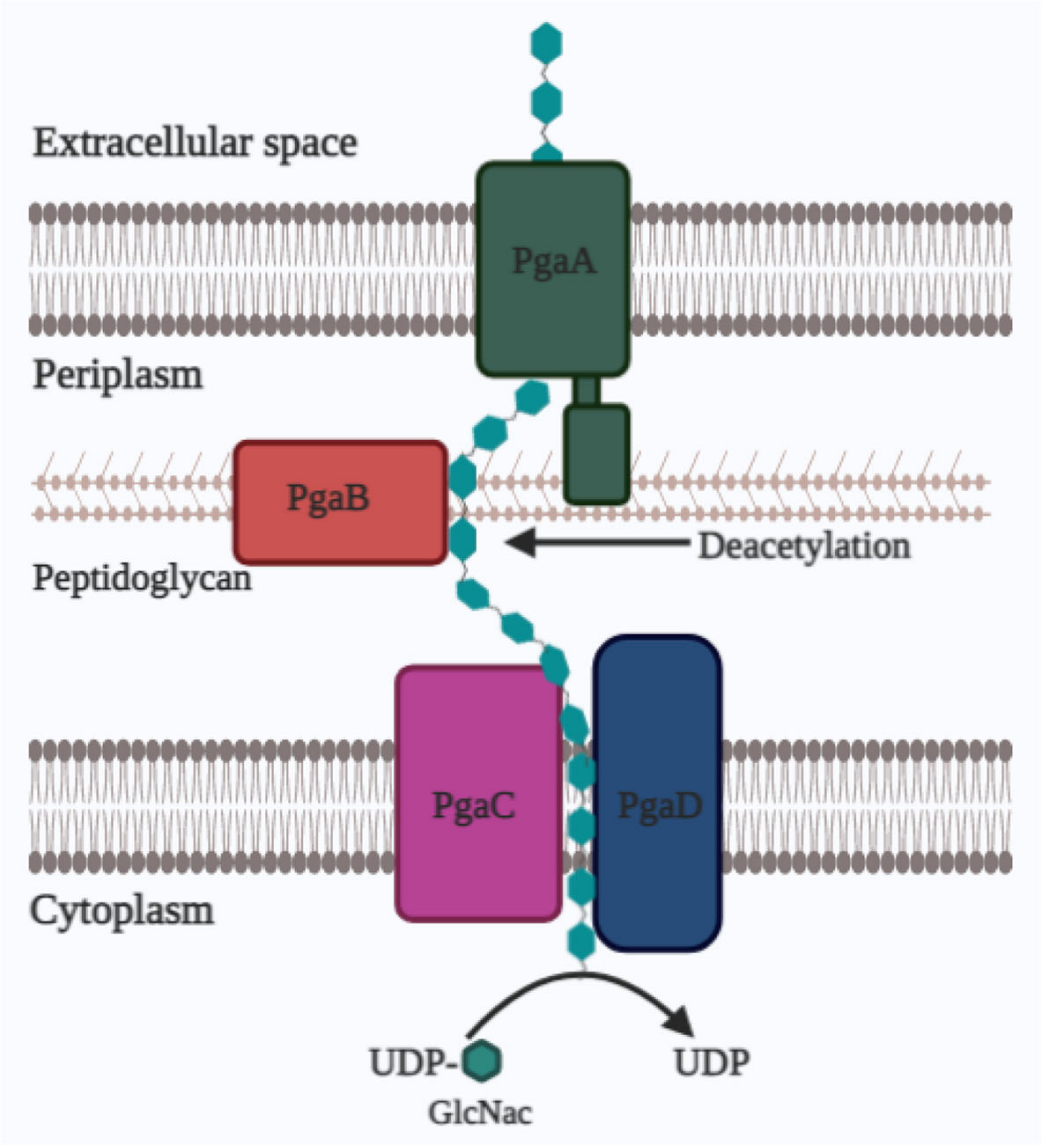
Biosynthesis of PNAG polysaccharide using pgaABCD operon

Bacterial species such as *S*. *aureus* and *P*. *aeruginosa* are considered as opportunistic pathogens that are known to cause many chronic infections, due to their resistance to several antibiotics and the formation of biofilms. As we have already seen, the three EPS molecules produced by *P*. *aeruginosa* strains are alginate, Pel, and Psl [40, 41]. Among these EPS molecules, Pel and Psl are the important EPS produced during the early colonizing, in non-mucoid isolates, and they play a different function in biofilm formation, antibiotic resistance, and immune evasion. In some cases, overproduction of both Pel and Psl leads to the formation of hyper-aggregative small colony variants (SCVs). Billings et al. showed that depletion of Psl makes the biofilm susceptible to range of antibiotics mostly young biofilms since Psl is important for initial attachment [40]. Pel polysaccharide provides resistance to biofilms against aminoglycosides [41, 42] and can confer tolerance to polymyxins, aminoglycosides, and fluoroquinolone antibiotics that too for the shorter duration [43]. Psl physically depletes the antibiotics and provide resistance to different antibiotics such as tobramycin, colistin, ciproflaxin, ceftazidime, piperacillin, gentamicin, imipenem, and polymixin B and also to the non-ionic surfactant polysorbate-80 [33, 44]. Whereas alginate is known to contribute to biofilm formation and render resistance to mucoidal isolates. It protects mucoidal *P*. *aeruginosa* from several antibiotics, DNase, as well as cationic AMPs by scavenging reactive oxygen species (ROS) and helps to escape host immune system. Therefore, it is crucial for the stability and persistence of mucoidal strains [40] in cystic fibrosis patients for decades. Also, it protects biofilm cells by sequestering the antimicrobials such as tobramycin [2]. *Staphylococci* strains are one of the primary causes of nosocomial infections, specifically associated with infections on implanted medical devices and catheters. *Staphylococcal* biofilms mainly consist of PNAG or PIA EPS. In the case of *S*. *epidermis*, PIA protect by reducing the ability of phagocytes and from antimicrobial peptides [45] (Fig. 2).

### Blocks deposition of complement components

**Fig. 3:**
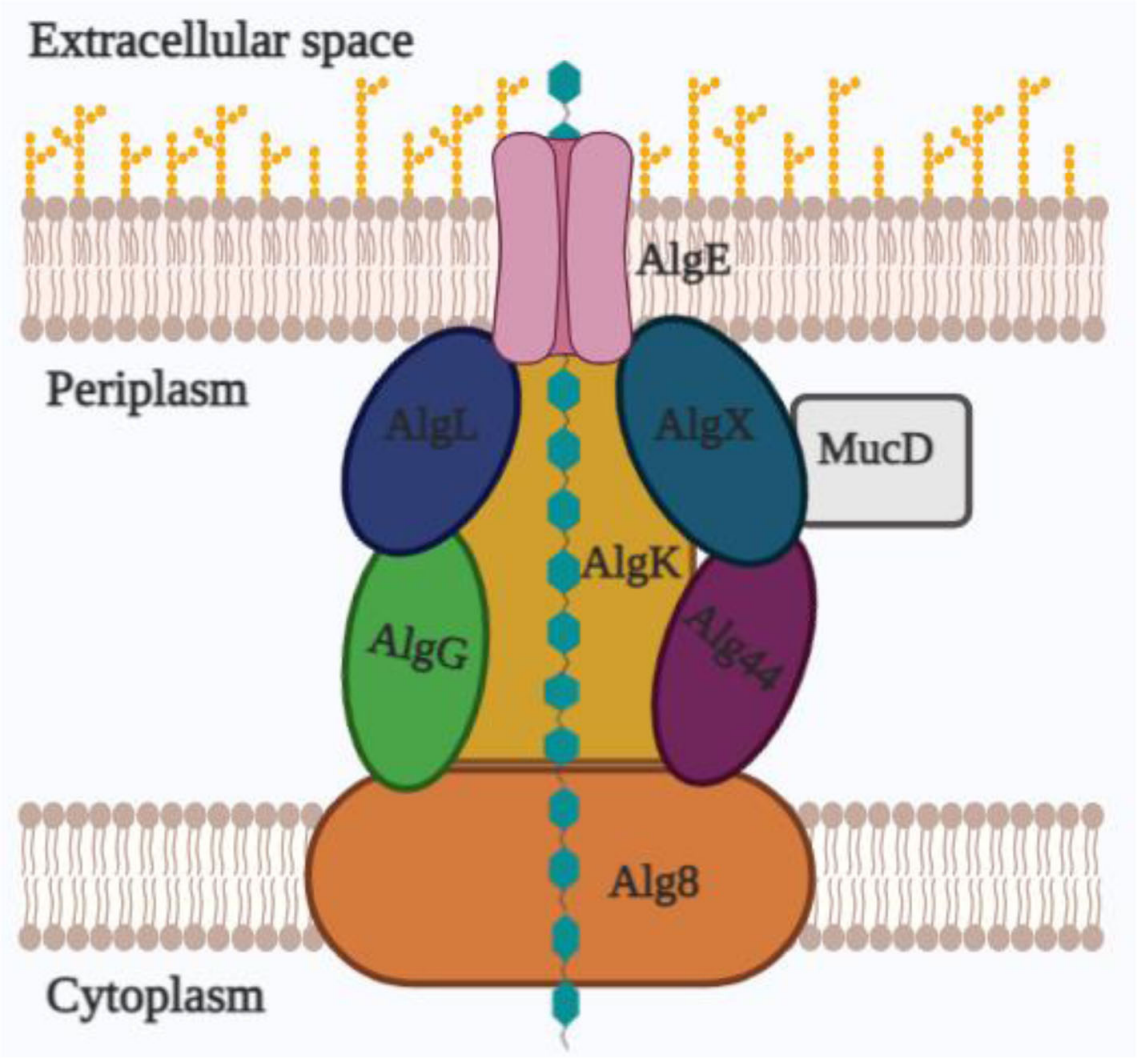
Biosynthesis of bacterial alginate

The complement system, a multifunctional group of circulating proteins, is a key component of innate immunity that recognizes pathogens through chemotaxis and opsonization. Complement once activated deposits proteins on the bacterial surface and promotes lysis of that particular bacteria. However, the EPS produced by the bacterial biofilms protect the cells from the complement components of the immune system [38]. For an example, Psl produced by *P*. *aeruginosa* block the deposition of complement on the bacterial surface; consequently, this provides an advantage to biofilms and helps to avoid immune attack. Along with these benefits, it reduces the chance of phagocytosis, the release of ROS, and cell attack by neutrophils [42, 46]. Since neutrophils can only attack pathogens smaller than 10 mm, so the pathogens habituating in biofilms have an advantage and can escape the attack. Also to gain access to biofilm, neutrophils first need to break the biofilm matrix but fail to do so as it can only exert pressure up to 1 Pka during phagocytosis [9, 43, 46]. Alginate produced in mucoid isolate protects biofilm from leukocyte phagocytosis and killing [6]. It has also been shown to scavenge hypochlorite, reduce polymorphonuclear chemotaxis, and also impede activation of complement [47, 48]. EPS produced by *Kingella kingae* PAM galactan, a galactofuranose homopolymer, provides resistance to serum killing by preventing opsonin deposition and evading complement-mediated killing. The functions of EPS depend on the polymer structure and composition [49] (Fig. 3).

### Overproduction of Exopolysaccharides

Generally, EPS are produced under antibiotic pressure or stressful conditions, since this is one of the mechanisms adapted to protect cells against different micro-organisms or antimicrobial agents [50]. Sub-minimal inhibitory concentrations (sub-MIC) or ineffective doses of antibiotics can induce antibiotic resistance or tolerance by increasing biofilm formation and altering biofilm matrix production. The presence of sub-MIC might influence bacterial gene expression, QS, virulence, and biofilm formation. Many studies have shown that sub-MIC of antibiotics can result in increased production of EPS like in the case of *S*. *aureus*, azithromycin increases the biofilm matrix production as well as sub-MIC of lactams led to increased e-DNA, and autoaggregation in biofilms. Antibiotics such as erythromycin, tetracycline, and quinpristin-dalfopristin when used at sub-MIC enhance ica genes expression in *Staphylococcus epidermidis*, which results in increased EPS production. In *P*. *aeruginosa*, sub-MIC tigecycline increased PNAG production and also the aminoglycoside, sub-MIC of impenem enhances the production of alginate and thus renders antibiotic resistance [36]. The work done by M. Cunha et al. revealed that the overproduction of alginate results in the formation of mature and thick biofilms. The formation of mature biofilms enhances the resistance and bacterial survival in lung infections since it can greatly tolerate host immune defenses and antibiotic treatment than planktonic cells [51]. Inactivating mutations in mucA gene also results in the overproduction of alginate in the biofilm matrix [48]. In some cases, overproduction of alginate has been shown to protect biofilm from certain antibiotics, also from protozoan eating *P*. *aeruginosa* biofilms [47, 48].

In the case of *Acinetobacter baumannii* biofilm-related infections, capsule structures in the biofilm matrix have been found responsible for tolerance to antibiotics such as AMPs. Also at sub-MIC, the transcriptional inhibitors such as Cm and Em cause rapid and reversible production of capsular EPS, thus conferring resistance to antibiotics as well as complement-mediated serum killing. One of the reasons for overproduction of EPS is the activation of BfmRS TCS. Capsular EPS also blocks the deposition or access of the serum complement components required for lysis. A study carried out by Vazquez-Rodriguez et al. showed that *Rhodotorula mucilaginosa* UANL-001L yeast strain was able to produce EPS under metal-stress conditions, and also when co-cultured with *E*. *coli* result in increased EPS synthesis [50].

**Fig. 4:**
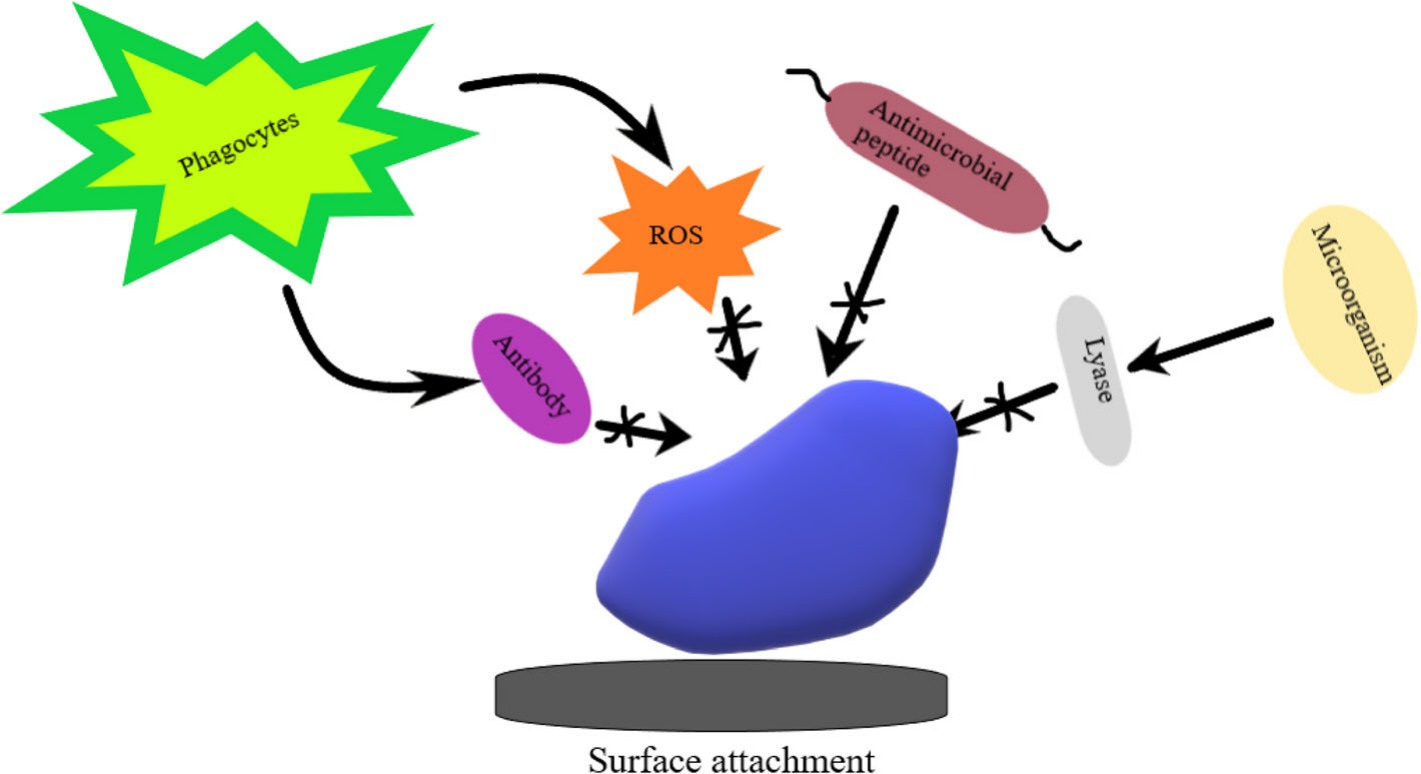
Bacterial biofilms exhibiting resistance due to modification in the exopolysaccharide

Oxidative stress is one of the conditions that induce the formation of biofilm. When exposed to oxidative stress, alginate production increases within the *P*. *aeruginosa* biofilm and thus overproduced biofilms protect from oxidative radicals [32]. ROS produced during aminoglycoside treatment stimulates the formation of biofilms in *P*. *aeruginosa* and *E*. *coli*. Iron used in the Fenton reaction stimulates the biofilm formation in *E*. *coli*, and sometimes the redox-active phenazine pyocyanin produced by *P*. *aeruginosa* also stimulates the biofilm formation. Oxidative stress, hypoxia generated by the host as defense, lead to drug-tolerant and persister cells in *Mycobacterium tuberculosis* biofilms. A. Trivedi et al. showed that reductive stress triggered by dithiothreitol (DTT) results in the formation of biofilm in *M*. *tuberculosis*. It was shown that reductive stress leads to envelope stress response wherein the envelope and periplasmic proteins unfolds and results in downregulation of ribosome biogenesis and translation machinery. Another way is that cellulose polysaccharide produced by bacteria combines with host polymers and create a complex and long-lasting shelter from leukocytes [52] (Fig. 4).

### Heterogeneity

**Fig. 5:**
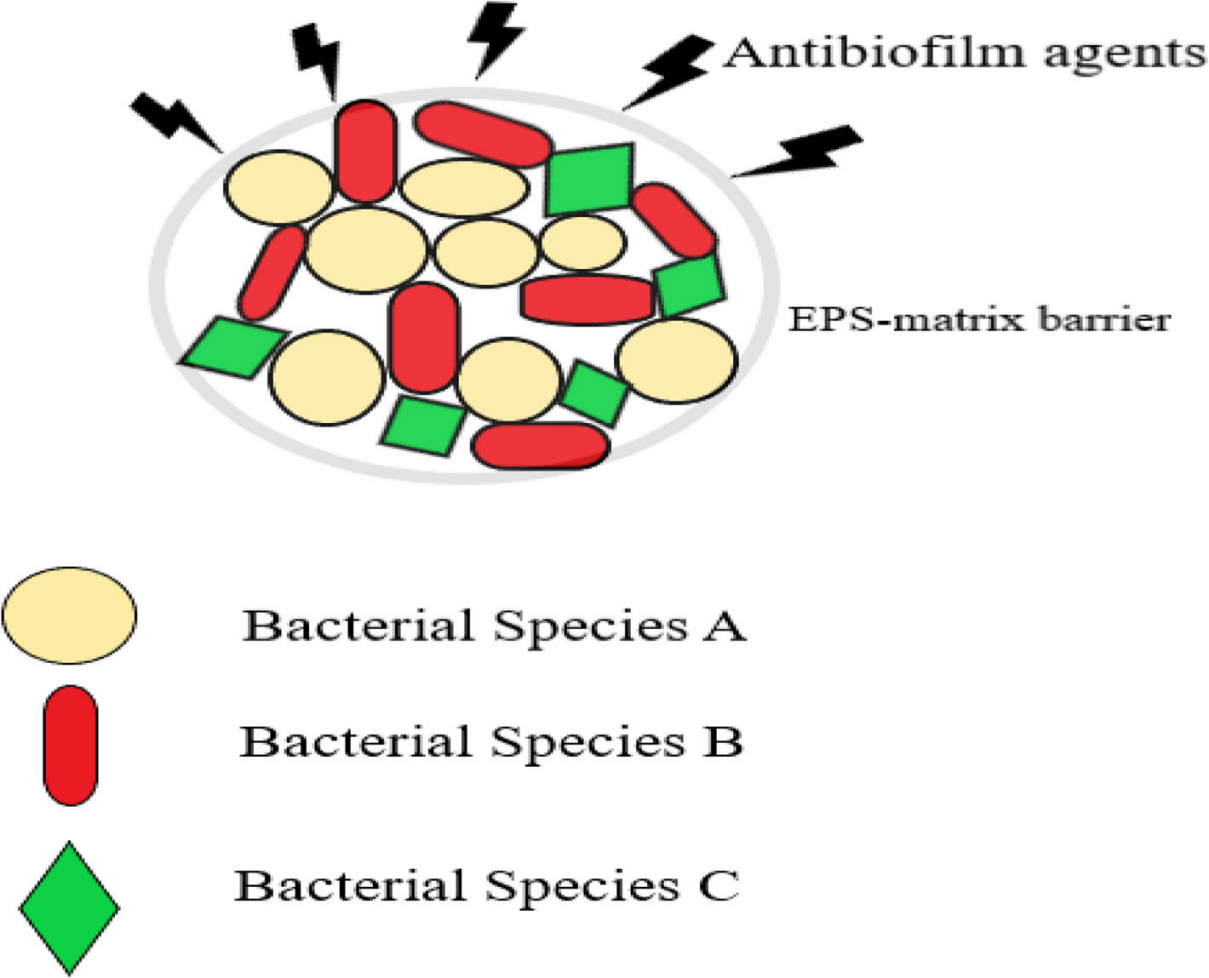
Representation of multiple bacterial species residing within the same biofilm

The polymicrobial nature of biofilms and proximity between bacterial species helps the non-resistant bacteria to tolerate antibiotic treatment relative to biofilms formed by an individual species. EPS and other biofilm components that segregate the antibiotics can provide resistance to other species that lacks this property, mostly via horizontal transfer of resistance conferring genes. For example, *Candida albicans* EPS, β-1,3-glucan, can bind to ofloxacin; so, when C. *albicans* present with other bacteria such as *E*. *coli* increases the tolerance to ofloxacin for *E*. *coli* biofilms. Likewise, when *S*. *aureus* present within *C*. *albicans* biofilm can resist vancomycin treatment. Psl produced in *P*. *aeruginosa* biofilm confer resistance to non-Psl producing bacteria against colistin [6, 36]. Bacterial species such as *E*. *coli* and *S*. *aureus*, which are incapable of synthesizing Psl, can be provided with resistance if co-cultured in biofilm with bacteria expressing Psl [40]. Periasamy et al. showed that in polymicrobial biofilm made up of *P*. *aeruginosa*, *P*. *protegenes* and *Klebsiella pneumonia*, Psl polysaccharide produced by *P*. *aeruginosa* protected other two pathogens, while the alginate and Psl mediate surfactant tolerance such as SDS in the community [32]. A study carried out by Perez et al. demonstrated that in polymicrobial infection with *Moraxella catarrhalis* and *Streptococcus pneumonia*, *M*. *catarrhalis* using quorum signal confer passive resistance to *S*. *pneumonia* against b-lactam antibiotics and bacterial clearance, whereas *S*. *pneumonia* provided protection to *M*. *catarrhalis* from azithromycin killing [53] (Fig. 5).

Polymicrobial interactions between *C*. *albicans* and *Streptococcus mutans* have been known to be associated with early childhood caries, which modifies the biofilm environment by enhancing the production of EPS and also increases the bulk of the biofilm. So, in this study, *C*. *albicans* stimulates the expression of glucosyltransferase B (GtfB), responsible for the formation of α-glucans biofilm matrix and its adhesion to the surface. When treated with a combination of drugs, EPS produced by the bacteria enhances tolerance to anti-fungal drugs by producing a dense layer of the polymeric matrix surrounding the fungal cells and decreasing the drug uptake [54]. So, it can be concluded that polysaccharides produced by pathogens may play a significant role in enhancing the tolerance to stress or antibiotics within the polymicrobial community and provide additional protection.

Generally, the role of matrix components in mono-species biofilms is studied for their antibiotic tolerance, so it becomes challenging to understand the role of biofilm matrix in multispecies bacterial biofilm and thus it becomes difficult to treat bacterial biofilm in real. To treat multispecies biofilms, it is necessary to understand cell to cell interactions within biofilm to develop better strategies for the prevention of biofilm formation and increase the therapeutic values of typical antibiotics.

### Deacetylation of the exopolysaccharides

A broad range of microbial species produces hexosamine-rich EPS such as PNAG, a homopolymer made up of *N*-acetylglucosamine (GlcNAc), produced by *Staphylococcus* species, *Yersinia pestis*, *Bordetella* species, and *E*. *coli*; Pel produced by *P*. *aeruginosa* is made up of GlcNAc and *N*-acetyl galactosamine (GalNAc), and *β*-1,4-linked *N*-acetylmannosamine polysaccharide produced by *Listeria monocytogenes* consists of terminal *α*-1,6-linked galactose (Gal) residues*.* All these polymers with hexosamine residues are partially deacetylated by polysaccharide deacetylases and as result, exposed amino groups are protonated and thus renders polysaccharide polycationic property. Many studies have shown the production of deacetylated polysaccharide can confer resistance and tolerance to host defenses and antimicrobial agents. For example, loss of PNAG deacetylation makes *Staphylococcal* species prone to neutrophil phagocytosis. PNAG inhibits the deposition of the complement on the cell surface of pathogens such as *Bordetella* species.

These cationic EPS protect biofilms from charged antimicrobial agents through electrostatic repulsion and deplete anionic molecules within the biofilm matrix to decrease the penetration of the agents [39]. For example, deacetylated PNAG of *S*. *epidermidis* provides resistance to cationic LL-37 and *β*-defensin 3 AMPs through electrostatic interaction and also protects the biofilm integrity from the bactericidal activity of anionic human AMP dermcidin [6]. PNAG produced in *Aggregatibacter actinomycetemcomitans* confers resistance to the cationic detergent cetylpyridinium chloride and in *S*. *epidermidis* protects the biofilm from microbicidal action of vancomycin [55, 56]. Similarly, negatively charged polymers in the biofilm matrix interact with positively charged agents and result in delayed penetration [39]. Extracellular enzymes such as b-lactamases, formaldehyde lyase, and formaldehyde dehydrogenase trapped in biofilm matrix inactivate positively charged and hydrophilic antibiotics and prevent their entry into the biofilm. At times biofilm matrix may act as an ion-exchange resin to prevent the entry of highly charged or highly chemically reactive agents into the deeper zones of biofilm, thus reduces their antibacterial activity [32]. Colanic acid, the EPS produced by *E*. *coli*, provides negative charge to outer cell surface and thus acts as a shield which prevents the entry of protons into the cells. This EPS act as protective barrier and enhances the pathogen survival in acidic condition like that of the human stomach [57].

Mechanism for biofilm-associated resistance to AMPs mostly involves its interaction with EPS in the biofilm matrix. As seen earlier, the positively charged EPS can prevent entry of charged AMPs in the biofilm through electrostatic repulsion. For an example, PIA the polysaccharide produced by *S*. *epidermidis* and *S*. *aureus* protect the biofilm cells from the actions of cationic. In *P*. *aeruginosa*, alginate when bind to AMPs can generate helical conformational for AMPs such as magainin II and cecropin P1, which is kind of similar to their interactions with the cytoplasmic membranes. So it can be concluded that alginate even though they are hydrophilic in nature they can mediate hydrophobic interactions with the following AMPs [6].

### Connection between exopolysaccharide and quorum sensing systems

*P*. *aeruginosa* produces several EPS such as alginate, Pel and Psl, PelD, PilZ, FleQ, and BcasA/BcsB. *P*. *aeruginosa* uses its QS system in the early stages of biofilm development, where it initiates the differentiation of individual cells into a complex multicellular biofilm. Two LuxI/R pairs (LasI/R, RhlI/R) which function in tandem to control the expression of virulence factors exist in this bacterium [10]. The *las* and *rhl* QS system consist of the LuxRI homologs LasRI and Rh1RI respectively. The LuxI homologs LasI and Rh1I are important for the regulation of the synthesis of las and rh1 signals, *N*-(3-oxododecanoyl)-l-homoserine lactone (3OC12-HSL) (PAI1), and *N*-butanoyl-l-homoserine lactone (PAI2), respectively. At low population density, LasI induces low production of 3OC12-HSL, whereas at high population density LasR binds its cognate AHL AI, and together they bind at promoter elements enhancing the production of virulence factors that are responsible for host tissue destruction during initiation of the infection process and also activates the transcription of *lasI*, *rhlR*. PAI1 is known to activate the Lux-R type transcription factor LasR and as a result, LasR-PAI1 triggers the production of various virulence factors such as LasB elastase, LasA protease, alkaline protease, exotoxin A, and LasR1. The las QS system is required for the biofilm development, so *P*. *aeruginosa lasI* mutants do not develop into mature biofilms and are completely flat and detergent sensitive and the *rhlI* mutant formed a colony that appeared hyper-wrinkled. Las QS system stimulates the transcription of the *pel* genes and also influences biofilm formation by *P*. *aeruginosa* PA14. PAI2 together with cognate regulator Rh1R enhances the production of secondary metabolites such as rhamnolipid, pyocyanin, LasB elastase, hydrogen cyanide, and cytotoxic lectins. The *las* QS system regulates the *rh1* QS system both at the transcriptional and post-transcriptional levels, as both these QS systems comprised hierarchical cascade and any mutation in QS can result in reduced virulence and thus reduces the pathogenicity [8, 22, 58, 59].

An intracellular signaling molecule 2-heptyl-3-hydroxy-4-quinolone (PQS) induces rh1I and the production of *lasB* requires the cooperation of PQS and NAHL. PQS is synthesized at the late stationary phase and it determines the interaction between the las and rh1 irrespective of the population density [22, 31]. PQS in the bacterial cell produces diketopiperazines which bind and activate LasR QS system. This phenomenon has high biological and pharmacological effects on cells of higher organisms, suggesting their role in the bacterial conversation with plant and animal cells rather than with other bacteria [22].

*S*. *aureus* uses a QS system with auto-inducing peptides (AIPs) as signaling molecules to regulate the expression of several virulence factors as well as the synthesis of AIPs. In a *S*. *aureu*s, only one copy of the QS system, i.e., *agr* system will be present on the chromosome, but the system can be any one of four types, each with the same basic components. The difference in each system will result in the formation of unique AIP signal such as AIP-I, AIP-II, AIP-III, and AIP-IV, and also cause corresponding changes in AgrB and AgrC to build and detect this signal, which is sometimes referred as the variable region. At high population cell density, signaling molecules produced and accumulated in the extracellular space are recognized by the bacteria and results in alterations in their transcriptional behavior. The Agr system is encoded by a 3.5-kb locus on the chromosome and consists of two transcriptional units, RNAII and RNAIII, driven by the two promoters P2 and P3, respectively [18, 25, 60]. One of the transcriptional units, RNAII transcript, harbors the *agrBDCA* genes; these genes are known to encode proteins required for AIP biosynthesis, transport, signal perception, and subsequent regulation of target genes. First, the signaling transduction starts with the transcription and translation of AgrD, which is the pro-peptide precursor of AIP. Secreted AgrD is post-transcriptionally modified into the final peptide by AgrB, which is an integral membrane endopeptidase, and is also modified via the type I signal peptidase SpsB. At high AIP concentration, AIPs bind to membrane-bound histidine kinase AgrC; this process further leads to auto-phosphorylation and initiation of the signal transduction. Once the signal cascade is initiated, AgrC further phosphorylates AgrA, which triggers the expression of RNAIII from the promoter P3. RNAIII controls the upregulation of genes encoding for proteins such as toxins and exoenzymes, also it downregulates the expression of genes encoding for surface associated adhesins. However, RNAIII transcript is responsible for the regulation of the majority of the genes; there are some genes which are directly regulated by AgrA. Among these, the most prominent is the direct regulation of RNAII from the P2 promoter, leading to upregulation of AIP production and a positive feedback loop. The psm and psm transcripts are regulated by AgrA as well as the hld gene for toxin is encoded within RNAIII. QS system activity is low at the initiation of *S*. *aureus* biofilm formation since Agr QS system can repress the expression of surface associated proteins such as FnBPs and SdrC, which are important for cell attachment. Agr QS system is activated in local patches instead of uniform expression and results in detachment of bacterial cells; this Agr QS activity creates a void in the biofilm which is later filled by regrowing cells in those areas. This cell detachment process is characteristic of *S*. *aureus* biofilms. Upregulation of Agr-related proteases by the Agr QS system is a consequence of the negative regulation of Rot via the RNAIII effector, whereas the core genome-encoded PSMs are directly regulated via AgrA. Therefore, activation of the Agr QS system leads to upregulation of PSMs and matrix-degrading enzymes, which are responsible for biofilm structuring and detachment of staphylococcal cells from the mature biofilm. As a result, inactivation of the Agr QS system enhances biofilm formation on certain surfaces by obstructing or delaying the dissemination process of the biofilm [25].

*Staphylococcal* adherence to biotic surfaces is mediated by cell wall anchored (CWA) proteins such as the fibrinogen-/fibronectin binding protein such as FnBPA and FnBPB and clumping factors A and B of *S*. *aureus.* ECM consists of large water filled channels, which accumulate antibiotic degrading enzymes such as β-lactamases and plays role in the adaptive resistance mechanisms due to e-DNA [60].

Sensor kinases recognize host signals such as human defensins and regulates the expression of various immune-stimulatory exo-proteins important for pathogenesis and biofilm generation, among them hemolysin (Hla), the MSCRAMMs FnbA and FnbB, and the extracellular thermonuclease Nuc are well studied. Fibronectin-binding proteins are important for biofilm formation at the level of intercellular accumulation, allowing cells to bind together via homophilic interactions between surface proteins, while Nuc is known to cleave e-DNA, a structural component of *S*. *aureus* biofilms. An early biofilm dispersal event, termed exodus, that is part of the five-phase biofilm model, takes place before tower formation in *S*. *aureus* biofilm maturation and is independent of Agr QS. With a set of elegant experiments, the expression of Nuc preceded the exodus event, which likely led to the degradation of e-DNA in the biofilm matrix. The exodus event could directly be linked to the 2CSTS system, as neither a sensor kinase nor a nuc mutant exhibited the exodus phenotype. As a result, it was found that *nuc* transcription was regulated by the 2C system in their biofilm model. Sensor kinases show polymorphism across *S*. *aureus* strains. For example, a point mutation in sensor kinase present in *S*. *aureus* Newman leads to hyperactivity of the SaeRS TCS, which was linked to the inability of this strain to form robust biofilms. In a recent study, it was also determined that several 2CSTS-regulated virulence genes in *S*. *aureus* are stochastically expressed in a subpopulation of cells in the nascent biofilm with an expression pattern matching the one of the thermonuclease Nuc. In *S*. *epidermidis*, mutation of *saeRS* leads to enhanced biofilm formation concomitant with higher Aap expression and e-DNA release. The derepression of SasG, a surface protein that promotes intercellular interactions and was implicated in the abiotic surface attachment [25, 60].

When the level of the AIP signal is controlled, the entire biofilm could be dispersed and resensitized to antibiotics. This mechanism is highly conserved across strains and even functions in the emerging *S*. *aureus.* The *agr* regulatory system controls a number of toxins and enzymes and also identifies the exact agents responsible for biofilm dispersal. Many studies have described that surfactant properties of δ-toxin can be responsible for the cell dispersal. δ-toxin was part of a larger family of PSM peptides, so multiple PSMs are critical in modulating *S*. *aureus* biofilm structure. Hence, most of the clinical isolates of *S*. *aureus* can make biofilms that are protease-labile, and also secretes many extracellular proteases that are known to self-cleave surface adhesins [25].

### Connection between exopolysaccharide and efflux pumps

A connection between the EPS diffusion barrier or the cell membrane and a multi-drug resistance (MDR) pump in providing resistance or tolerance to antibacterial agents has been observed in several bacterial pathogenesis [38]. Drug efflux is one of the mechanisms responsible for antibiotic resistance by in both Gram-negative and Gram-positive bacteria. One study involving *S*. *mutans* showed that expression of efflux transporter encoding genes increased when cultured with sub-MIC of tetracycline, also when exposed to heat shock or osmotic stress. Liu et al. reported that when *S*. *mutans* treated with sub-MIC of chlorhexidine (CHS) resulted in increased expression of differentially expressed efflux pump genes *lmrB* in order to survive in adverse environment*.* Inactivation of this efflux pump may result in overexpression of other homolog efflux pumps to compensate for the loss of *lmrB* as well as significant increase in the biofilm biomass as a consequence of enhanced thickness and EPS production was observed [15]. When *E*. *coli* treated with fluoroquinolones resulted in increased efflux and decreased penetration of the agent [29]. It can be concluded that inactivating efflux pumps does not increase the antibiotic susceptibility, since sub-MIC can stimulate various alternative mechanism which can be adapted to compensate for the loss and help bacteria to survive antibiotic pressure.

In *A*. *baumannii*, MDR pumps such as AdeABC, AdeFGH, and AdeIJK belonging to RND superfamily play an important role in biofilm formation. So, when exposed to sub-MIC of meropenem and tigecycline resulted in overexpression of *adeB* and *adeG* genes and increased biofilm formation. Also elevated expression of *adeB* and *adeG* genes is positively correlated with transcription level of *adeI* gene, thus displaying relation between the RND efflux pumps and QS. Also, the increased expression of AdeABC and AdeFGH transporters induces the export of AHLs which lead to increased biofilm formation [61]. In *P*. *aeruginosa* mutant lacking MexAB-OprM efflux pumps, reduced biofilm formation as well as reduced production of QS-controlled virulence factors have been observed due to intracellular accumulation of 3OC12-HSL, an important AHL that induces QS [18, 31]. When *P*. *aeruginosa* treated with azithromycin showed reduced biofilm formation as a consequence of reduced production of 3OC12-HSL and *N*-butyryl-l-homoserine lactone (C4-HSL). So when both the molecules were added in the presence of azithromycin, it resulted in recovery of biofilm formation, thus confirming their role in biofilm formation [18, 20]. In *S*. *aureus*, three genes which code for MFS efflux pumps, i.e., NorB and NorC pumps are known to export substances such as cetrimide, ethidium bromide, quinolones, and tetraphenylphosphonium, whereas MdeA pump export wide range of quaternary ammonium compounds and antibiotics. Acid shock and reduced aeration results in overexpression of NorB to protect biofilms [18].

Several MDR pumps encoding genes belonging to RND superfamily have been known to play a role in antibiotic resistance in *P*. *aeruginosa*. Out of all the known efflux pumps, MexAB-OprM, MexCD-OprJ, MexEFOprN, and MexXY that have been mostly studied in *P*. *aeruginosa* [32] are resistant to drugs like tetracycline, chloramphenicol, quinolones, β-lactams, and these drugs are exported by the MexAB-OprM efflux pump [3]. MexAB-OprM and MexCD-OprJ efflux pumps are important for biofilm formation in the presence of azithromycin. Likewise, MexAB-OprM efflux pump provide resistance against colistin [4]. Another is the AcrAB-TolC efflux system belonging to RND superfamily is found in *E*. *coli* and is known to export substrates such as chloramphenicol, fluoroquinolones, fusidic acid, rifampicin, tetracycline, ethidium bromide, bile salts, etc. These efflux systems are overexpressed in bacterial biofilms and due to exposure to several antibiotic agents [3]. Overexpression of efflux pumps is an adaptive mechanism used by bacteria to overcome an antibiotic pressure, as efflux pumps play role in the biofilm formation and it can provide resistance associated to biofilms [20]. Overexpression of more than one efflux pump such as MexAB-OprM and MexXY efflux pumps can confer multi-drug resistance in bacteria like *P*. *aeruginosa* [9]. *P*. *aeruginosa* biofilm-related infections may show resistance to fluoroquinolones as a result of mutations in the genes encoding DNA gyrase, topoisomerase IV, or due to overexpression of efflux pumps which cause accumulation of antibiotics in the bacterial cell [30]. Efflux pumps show broad range of substrate specificities, for example, AcrAB-TolC efflux pumps in *E*. *coli* export chloramphenicol, fluoroquinolone, tetracycline, novobiocin, rifampin, fusidic acid, nalidixic acid, and b-lactam antibiotics. On the other hand, the AcrAB-TolC efflux pump in *S*. t*yphimurium* exports antimicrobial agents such as quinolones, chloramphenicol, tetracycline, and nalidixic acid. In the case of *P*. *aeruginosa*, MexAB-OprM efflux pumps which are the homolog of AcrAB-TolC and MexXY-OprM efflux pumps together export fluoroquinolones, tetracycline, and chloramphenicol [28].

### Secretion systems

One of the techniques used by most Gram-negative bacteria to survive in a mixed-species biofilm is the type 6 secretion system (T6SS). The T6SS is like a contact-dependent system which does not require a specific receptor to target cells and thus deliver toxic effector into surrounding cells. This system provides an advantage to bacteria like *V*. *cholerae* to target phagocytic cells or bacterial cells hampering their growth and confer tolerance. There are few bacteria like *P*. *aeruginosa* which have to adapt a unique mechanism to tackle T6SS attacks by constructing its own T6SS apparatus. It was proven that a layer of EPS could act as a shield or physical barrier and protect the bacterial colonies from exogenous T6SS attacks. The defense mechanism by EPS depends on its structure like if the EPS consists of open channels that are perpendicular to the cells; the ability to block the attack would depend on the angles of the attacks. Another structure would be the presence of small gaps in the EPS layer, where the T6SS apparatus is localized at the gaps [62]. This type of defense mechanism can be useful when a bacterium is present in the biofilm consisting of predatory T6SS producing bacterial cells and protect from exogenous attacks.

A connection between MDR efflux pumps and expression of the type III secretion (T3SS) system which has been implicated in several bacterial pathogenesis has been described. Continuous overproduction of MexCD-OprJ or MexEF-OprN efflux pumps in *P*. *aeruginosa* affects the expression of the T3SS genes negatively, as a consequence of reduced expression of the exsA gene that encodes for the transcriptional activator of T3SS [32]. Another case involving *Salmonella enteritidis* MDR mutants revealed that reduced expression of invasion genes in the SP-1 region encoding a T3SS and genes responsible for flagella biosynthesis reduced the ability of the bacterial cell as well as its invasion capability thus hampering the bacterial adherence [20].

## Strategies to treat biofilm related infections

Once biofilms are established, it becomes difficult to get rid of it. The main purpose of any research should be to find new molecules to target the biofilm mode of growth to prevent and treat chronic bacterial infections. Since EPS are known to protect against antibiotics and the immune system, the main focus should be on developing drugs that could weaken or disrupt the biofilm by targeting the EPS production and other extracellular matrix components.

### Using exopolysaccharide repressors

High-throughput screening (HTS) approaches are being applied to look for new molecules which could reduce biofilm formation, and which could also possibly detach preformed biofilms in many species of bacteria. Van Tilburg Bernardes et al. found Pel repressors that could reduce the expression of the Pel genes in *P*. *aeruginosa* biofilms and as a result, they were capable of reducing biofilm formation. These compounds also showed anti-virulence activity. Out of all the compounds studied, acetylcholine, a known anti-biofilm compound, was confirmed repressor and there were compounds which were structurally similar and shared benzothiazole component. These compounds did not inhibit the bacterial growth but reduced the expression of Pel gene, thereby affecting the biofilm growth, and thus can be used for treating *P*. *aeruginosa* infections. Causing mutations in EPS expressing genes can reduce the ability to form a biofilm, by reducing the bacterial cell adherence and expression of EPS in the biofilm [43].

### Using exopolysaccharide degrading enzymes

Mainly detachment process is carried out by sing EPS degrading enzymes such as glucanohydrolases, dispersin B, or DNAse to eradicate biofilms [16] have shown promising results. These degrading enzymes facilitate the diffusion of antibiotics into the biofilm and also can be used in combination with antimicrobial agents, as a successful strategy to treat established biofilm-related infections [63]. In *P*. *aeruginosa*-related infections PslG and PelA, a glycosyl hydrolase can prevent biofilm formation as well as disrupt already established biofilms from a broad range of *Pseudomonas* strains at nano-molar concentrations [42]. So pre-treatment with PslG reduces the MIC of antibiotics such as tobramycin and ciproflaxin, which can result in the eradication of already established biofilms [36]. Degrading Pel polysaccharide makes the *P*. *aeruginosa* biofilm prone to colistin treatment [55]. Another enzyme Dornase alfa which is a DNAse I is used to disrupt *P*. *aeruginosa* biofilms as it hydrolyzes the e-DNA with the extracellular matrix [42]. The biofilm detachment process involving the PIA polysaccharides are degraded by the action of PIA-degrading detergents like peptides, which disturbs the non-covalent interaction between the bacterial cell surface and PIA [2]. The anti-biofilm peptides like cathelicidin; dispersal signals such as nitric oxide and *cis*-2-decenoic acid; anti-matrix molecules, or surfactant molecules such as chitosan, rhamnolipids, and urea; and sequestration molecules like EDTA and lactoferrin are used for surface detachment of the biofilms [16]. This makes it important to understand the composition of biofilm matrices such as EPS and its interaction with different degrading enzymes and antimicrobial agents. This way an effective combination of degrading enzymes and antimicrobial agents in combination can be used to eradicate already established biofilms.

### Combination therapy

Using different antibiotics in combination with biofilm disrupting drugs or antimicrobial agents has proven to be an innovative approach to treat biofilm-related infections. This way the effectiveness of antibiotics used can be enhanced and so together they could synergistically affect the biofilm formation as well as decrease the probability of resistance evolution. Nitric oxide known for disrupting *P*. *aeruginosa* biofilms can be used to in combination with antibiotics to treat chronic infections effectively [64].

#### In combination with AMPs

AMPs have shown profound effects when used in combination with typical antibiotics and thus can be used in many antibiotic treatments. AMPs are produced by many multicellular organisms and hence can target a broad range of metabolic processes of bacterial cells. Since AMPs consist of a net positive charge, it can interact with the negatively charged component of the biofilm and gain access to it. Once AMP enters into biofilm, they result in membrane disruption via various mechanisms, also the cell integrity is lost and these lead to cell death and it also has the potential to kill dormant cells residing deep inside the biofilms. AMPs also exhibit intercellular inhibitory activity through various important processes such as cell division, nucleic acid metabolism, cell wall biosynthesis, as well as can modulate the immune response stimulating cytokine production, acting as chemokines, and promote wound healing. Cationic AMPs like LL-37, piperacillin, buforin II, ceprocin P1, indolicidin, nisin, and magainin II have shown promising effects when used in combination with antibiotics such as polymyxin E, piperacillin, azithromycin, daptomycin, linezolid, and clarithromycin; it increases the bioavailability of antibiotic against many MDR Gram-negative bacteria as well as methicillin-resistant *S*. *aureus* (MRSA) pathogens. In MDR *P*. *aeruginosa*, AMP like synthetic cyclolipopeptide analog of polymyxin in combination with carbapenems kills the established biofilm [65]. Dispersin B, a polysaccharide hydrolyzing enzyme that degrades poly-*N*-acetylglucosamine, when used in combination with a silver-containing wound dressing, or with an AMP can synergistically affect chronic wound associated biofilm infections such as MRSA [36, 64]. AMP DP7 when combined with antibiotics such as azithromycin and vancomycin can effectively eradicate biofilms formed by highly antibiotic-resistant bacteria such as *S*. *aureus*, *P*. *aeruginosa*, and *E*. *coli* [66]. AMP like PI has the potential to degrade the EPS produced by *S*. *mutans* and thus causing reduction in biofilm formation on surfaces like polystyrene or and saliva-coated hydroxyapatite. Another is the human liver-derived AMP hepcidin 20, which has the capability to reduce the mass of extracellular matrix and also can cause alterations in the biofilm structure of *S*. *epidermidis* by targeting PIA polysaccharide [6]

#### In combination with different exopolysaccharides

In rare occasions, a particular EPS produced by one bacterial biofilm has been shown to possess toxic activity against biofilm produced by some other bacterial species. This is one of the adaptive capabilities exhibited by bacteria to survive in a highly competitive environment. The use of biologically derived molecules with conventional antibiotics can be considered as a better therapeutic option. One such example is surfactant EPS (SEPS), a derivative of EPS, exhibits antimicrobial properties for a broad range of bacterial species in a non-specific manner. SEPS being anionic in nature binds to membrane cations, and disrupts cell membrane by altering the cell permeability. Sengupta et al. showed that SEPS extracted from *Ochrobactrum pseudintermedium* ciprofloxacin together gave rise to the possible highest antibacterial activity [67]. Bacterially derived metabolites by marine *actinomycetes* have been shown to have an anti-biofilm activity against *Vibrio* and *S*. *aureus*. A combination of pigment prodigiosin having antibacterial property and a surfactant EPS extracted from *Serratia marcescens* together act as an effective antimicrobial agent. Spanò et al. showed that EPS1-T14, a EPS produced by *Bacillus licheniformis* strain T14, possesses potential antibacterial activity and can inhibit the formation of biofilms by multi-resistant bacteria such as *E*. *coli*, *K*. *pneumoniae*, *P*. *aeruginosa*, and *S*. *aureus* [68]*.*

#### In combination with bacteriophages

Bacteriophages, a virus that infects bacteria, used in combination with typical antibiotics eradicate the biofilm formed by pathogenic bacteria such as *P*. *aeruginosa* and MRSA. The combinatory therapy including bacteriophage and ciprofloxacin effectively treat P. *aeruginosa* associated infections [36, 37]. T. Tkhilaishvili et al. show that pre-treatment with *S*. *aureus*-specific bacteriophage Sb-1 and subsequent treatment with antibiotics such as fosfomycin, rifampin, vancomycin, daptomycin, and ciprofloxacin was able to degrade the MRSA extracellular polysaccharide and target the persister cells. This combination can completely eradicate MRSA biofilm and thus can be used to treat biofilm infections [69].

#### In combination with compounds of nature origin

A new approach including the use of compounds of nature origin that could act against resistant bacteria is increasing. The natural product brazilin has been shown to have antibacterial activity against unrelated bacterium *Propionibacterium acnes*, and acetylene exhibits both anti-biofilm and anti-virulence activity against *Candida albicans* [45]. Abu El-Wafa et al. found that pomegranate and rosemary extracts when used in combination with conventional antibiotics such as piperacillin, ceftazidime, imipenem, gentamycin, or levofloxacin increased the efficacy of antibiotics against and resulted is synergy effects against *P*. *aeruginosa* biofilms, by significantly reducing MIC of antibiotics. These extracts inhibit biofilm formation due to the presence of polyphenol compounds which are known to disable QS system, suppress bacterial cell adherence and motilities as well as inhibited polymeric matrix synthesis [70]. Essential oils from parsley, lovage, basil thyme, and hemp lead to increased cell permeability, alterations in the bacterial cell membrane and cell wall, ATP loss, inhibition of protein synthesis, alterations in pH value, DNA damage, and inhibition of QS system in many bacterial species like *Bacillus cereus*, *S*. *aureus*, *P*. *aeruginosa*, *E*. *coli*, and *Salmonella enterica* serovar typhimurium [63].

Therefore, combining agents having some anti-bacterial potential with typical antibiotics not only enhance the efficacy of antibiotics but also reduces the MIC of the antibiotic used. This would reduce the side effects of high dosage as well as the development of antibiotic resistance.

### Using efflux pumps inhibitor

One of the promising strategies to treat bacterial biofilm infections is to use efflux pump inhibitors targeting broad range of efflux pumps that would inhibit the flux of substances from different bacterial species and as well as to increase the potential of the exported substances [18, 27]. The most studied bacterial species concerning to efflux pump and inhibitors is *P*. *aeruginosa* [27]. Efflux pump inhibitors such as thioridazine, Phenyl-Arginine β-naphthylamide (PAβN), or the 1-(1-naphthylmethyl)-piperazine (NMP) are known to affect biofilm formation in bacteria such as *E*. *coli*, *Klebsiella pneumonia*, *S*. *aureus*, and *P*. *putida* [3, 4]*.* The first identified inhibitor is PAβN which in combination with fluoroquinolones have shown inhibitory action against AcrAB-TolC, MexAB-OprM, MexCD-OprJ, and MexEF-OprN pumps [3]. Also using combinations of efflux pump inhibitors such as thioridazine and PaβN synergistically affects the biofilm formation in *E*. *coli* and *K*. *pneumoniae* [18].

### Using quorum sensing inhibitors

Many QS inhibitors have been discovered such as *N*-(2-oxocyclohexyl)-3-oxododecanamide which works antagonistically toward the las QS system; *N*-(2-hydroxyphenyl)-3-oxododecanamide is known to interfere with the rh1 QS system as well as las QS system, and *N*-octanoyl cyclopentylamide (C8-CPA) moderately inhibited expression of the *lasB*-*lacZ* transcriptional fusion gene and also the expression of lasB which encodes for LasB elastase in *P*. *aeruginosa* PAO1. Another inhibitor *N*-decanoyl cyclopentylamide (C10-CPA) which is a stronger QS inhibitor is known to interfere with the expression of virulence genes regulated by both las and rh1 QS systems by inhibiting the interaction between their response regulators and AIs. The length of the acyl side chain and the ring structure is critical for C10-CPA to act as an inhibitor. C10-CPA analogs with cyclopropylamide, cyclobutylamide, cyclohexylamide, cycloheptylamide, and cyclooctylamide rings exhibit little inhibitory activity [58]. Lactoferrin an ubiquitous and abundant constituent of human external secretions inhibits biofilm development by *P*. *aeruginosa.* Lactoferrin stimulates twitching which causes the bacteria to wander instead of forming biofilms [71].

Programmed cell death through autolysis has been observed in *P*. *aeruginosa* isolates and is an essential physiological event for subsequent differentiation and dispersal of a subpopulation of surviving biofilm cells. Cell death in this bacterium requires a functional rpoN gene which regulates two types of cell structures, i.e., type IV pili (T4P) and flagella. Water channels around *P*. *aeruginosa* micro-colonies are regulated by the QS-controlled production of rhamnolipid surfactants. Dispersal of the bacterial cells from voids present within the biofilm has been described. The LasR-dependent AI, *N*-(3-oxododecanoyl)-homoserine lactone, prevents the binding of the RhlI-dependent AI, *N*-(butryl)-homoserine lactone, to its cognate regulator RhlR. This control of RhlI/RhlR autoinduction by the LasI/LasR system assures that the two systems initiate their cascades sequentially and in the appropriate order additional AI has recently been demonstrated to be involved in QS in *P*. *aeruginosa*. This signal is noteworthy because it is not of the homoserine lactone class [71].

Apart from antibiotic treatment, it is also important to make strategies to prevent biofilm formation. One way is to modify the abiotic surface properties by layering the surface with antimicrobial agents which could inhibit the bacterial biofilm adhesion and development. For example, surfaces of the medical devices or surgical implants can be altered such as making the surface hydrophilic and incorporating antimicrobial agents such as copper, probiotics to prevent the bacterial biofilm adherence and also control the transmission of bacterial pathogens into the environment [37]. Bismuth thiols are known to have potent antimicrobial and antibiofilm activity against a broad range of bacterial species and have been used to treat biofilm-related infections. Bismuth thiols when used at sub-MIC reduce the biofilm formation or disrupt the already formed biofilms in MRSA, *P*. *aeruginosa*, and further can make bacteria susceptible to antibiotic treatment or host immune responses. As a result, efficiency of many antibiotics can be increased when used in combination with sub-MIC bismuth thiols. Medical implants coated with a metal such as silver are also efficient in inhibiting biofilm formation [36]. However, these coated antimicrobial agents are efficient only for a short duration and thus cannot be used for the long run, and these might generate multidrug-resistant bacteria.

## Conclusion

Biofilm formation is evolutionary adaptability gained by bacterial species to tolerate and survive in extreme conditions including mechanical stress and antibiotic treatments. Biofilm is an aggregation of micro-organisms that are irreversibly attached to a substratum and are enclosed in a self-produced extracellular polymeric substance composed of EPS, lipids, e-DNA, and proteins. Antibiotic resistance is a natural phenomenon observed in bacterial species. The formation of bacterial biofilms is considered one of the major reasons for several chronic infections. A deeper understanding of how biofilm is formed, the antibiotic resistance mechanism, and the interaction of the biofilms with the host are important. However, antibiotic treatment involving the use of conventional antibiotics is repeatedly proven to be inadequate to eliminate biofilm-related infections [72]. It has also been revealed the use of ineffective doses of antibiotics may promote biofilm formation and give rise to antibiotic-resistant cells. Therefore, it has become important to develop new approaches to combat antibiotic resistance and drug tolerance. The major component of the biofilm matrix is EPS which plays a major role in the maintenance of the biofilm structure, so targeting EPS is necessary for the complete eradication of biofilms. Using EPS degrading enzymes like polysaccharide lyases along with conventional antibiotics makes the bacteria susceptible to antibiotic treatment and thus helps in getting rid of the biofilms. A biofilm formed in general is mostly composed of several bacterial species populations; this property makes the antibiotic treatment ineffective which otherwise is found to be effective on biofilms formed by single species. In polymicrobial biofilms, the EPS producing bacteria has the potential to confer resistance to non-EPS-producing bacteria and proximity promotes the transfer of antibiotic-resistant genes. This gives rise to the emergence of studies involving cell-to-cell interactions within polymicrobial biofilms and also with different antimicrobial agents, and developing an effective antibiotic treatment that would work on the whole biofilm. Combinatory therapies targeting different components of the matrix have increasingly attracted attention due to its various benefits. Therapies including the use of antibiofilm and antimicrobial agents have expanded over the years due to their high potential and low cell toxicity. Currently, antibiotics used can decrease the number of biofilms formed and mostly for a short duration, but they cannot eliminate the biofilms formed. One of the major reasons is the formation of non-metabolically active cells deep in the biofilm since drugs can only act on metabolically active cells and not on dormant cells. So, there is a chance for relapses of infections after treatment because the dormant cells become active which was metabolically inactive becomes active and forms a new biofilm. In case of infections due to biofilms formed on implanted devices, the only way to treat is to completely remove the infected tissues or implanted devices which would be costly. Despite the development of several strategies to prevent biofilm formation, a proper and efficient approach is still not developed. The reason for the delay is that micro-organisms keep evolving dynamically when exposed for long to antibiotics and acquire several ways to tolerate antibiotic treatment. As per future perspective, the approach which would save time, make reasonable use of drugs, and reduce the healthcare costs is required. Another major gap in research that remains is the physical and mechanical characterization of these biofilms. Mechanical characterization of these biofilms and understanding the processes underlying how they trap drugs can bring novel breakthroughs in the field. This can lead to development of drugs that can penetrate the biofilm matrix and also the development of micro and nano carriers which can help the drugs penetrate the biofilm matrix. Also another important area of research should be to understand the role of extracellular genetic material better. Although previously it has not been characterized, small extracellular RNA that are packaged in extracellular vesicles may be an important step to understand the cell-to-cell communication in bacteria which helps to improve the fitness of the bacteria. New compounds and strategies should also be developed to disrupt and prevent biofilms.

## Data Availability

Not applicable.
